# Molecular Recognition of GalNAc in Mucin-Type O-Glycosylation

**DOI:** 10.1021/acs.accounts.2c00723

**Published:** 2023-02-23

**Authors:** Ignacio Sanz-Martinez, Sandra Pereira, Pedro Merino, Francisco Corzana, Ramon Hurtado-Guerrero

**Affiliations:** † Institute of Biocomputation and Physics of Complex Systems (BIFI), Glycobiology Unit, University of Zaragoza, Mariano Esquillor s/n, Campus Rio Ebro, Edificio I+D, 50018 Zaragoza, Spain; ‡ Department of Organic Chemistry, Faculty of Sciences, University of Zaragoza, Campus San Francisco, 50009 Zaragoza, Spain; § Department of Chemistry, Centro de Investigación en Síntesis Química, University of La Rioja, Madre de Dios 53, 26006 Logroño, Spain; ∥ Copenhagen Center for Glycomics, Department of Cellular and Molecular Medicine, University of Copenhagen, Copenhagen DK-2200, Denmark; ⊥ Fundación ARAID, 50018 Zaragoza, Spain

## Abstract

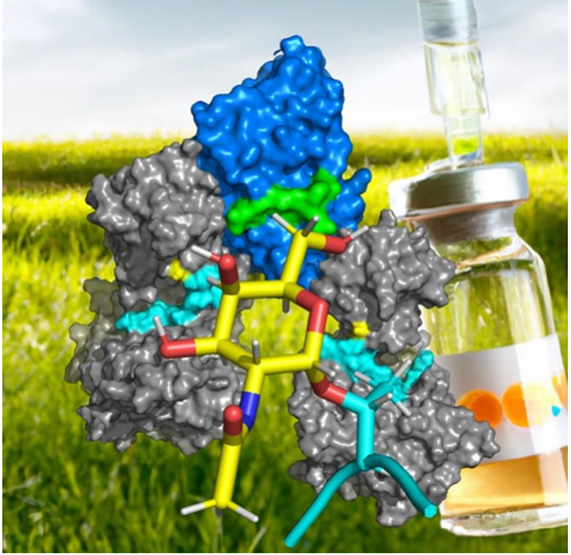

*N*-Acetylgalactosamine (GalNAc)-type O-glycosylation is an essential posttranslational modification (PTM) that plays fundamental roles in biology. Malfunction of this PTM is exemplified by the presence of truncated *O*-glycans in cancer. For instance, the glycoprotein MUC1 is overexpressed in many tumor tissues and tends to carry simple oligosaccharides that allow for the presentation of different tumor-associated antigens, such as the Tn or sTn antigens (GalNAc-α-1-O-Thr/Ser and Neu5Acα2-6GalNAcα1-O-Ser/Thr, respectively). In other cases, such as tumoral calcinosis associated with O-glycosylation of the fibroblast growth factor 23, *O*-glycans are absent or less abundant. Significant progress has been made in determining the three-dimensional structures of biomolecules that recognize GalNAc, such as antibodies, lectins, mucinases, GalNAc-transferases, and other glycosyltransferases. Analysis of the complexes between these entities and GalNAc-containing glycopeptides, in most cases derived from crystallographic or NMR analysis, provides an understanding of the key structural elements that control molecular recognition of these glycopeptides. Here, we describe and compare the binding sites of these proteins in detail, focusing on how the GalNAc moieties interact selectively with them. We also summarize the differences and similarities in GalNAc recognition. In general, the recognition of GalNAc-containing glycopeptides is determined by hydrogen bonds between hydroxyl groups and the *N*-acetyl group of GalNAc with proteins, as well as CH-π contacts in which the hydrophobic α-face of the sugar and the methyl group of NHAc can be involved. The latter interaction usually provides the basis for selectivity. It is worth noting that binding of these glycopeptides depends primarily on recognition of the sugar moiety, with some exceptions such as a few anti-MUC1 antibodies that primarily recognize the peptide backbone and use the sugar to facilitate shape complementarity or to establish a limited number of interactions with the protein. Focusing specifically on the GalNAc moiety, we can observe that there is some degeneracy of interactions within the same protein families, likely due to substrate flexibility. However, when all studied proteins are considered together, despite the commonalities within each protein family, no pattern can be discerned between the different families, apart from the presence of common residues such as Tyr, His, or Asp, which are responsible for hydrogen bonds. The lack of a pattern can be anticipated, given the diverse functions of mucinases, glycosyltransferases, antibodies, and lectins. Finally, it is important to point out that the conformational differences observed in solution in glycopeptides bearing GalNAc-α-1-O-Ser or GalNAc-α-1-O-Thr also can be found in the bound state. This unique characteristic is exploited, for instance, by the enzyme C1GalT1 to broadly glycosylate both acceptor substrates. The findings summarized in this review may contribute to the rational structure-guided development of therapeutic vaccines, novel diagnostic tools for early cancer detection, and new cancer treatments for cancer with tailored anti-Tn or anti-STn antibodies or new drugs to inhibit GalNAc-T isoenzymes.

## Key References

- de Las Rivas, M.; Paul Daniel, E. J.; Narimatsu, Y.; Compañón, I.; Kato, K.; Hermosilla, P.; Thureau, A.; Ceballos-Laita, L.; Coelho, H.; Bernadó, P.; Marcelo, F.; Hansen, L.; Maeda, R.; Lostao, A.; Corzana, F.; Clausen, H.; Gerken, T. A.; Hurtado-Guerrero, R. Molecular basis for fibroblast growth factor 23 O-glycosylation by GalNAc-T3. *Nat. Chem. Biol.***2020**, *16*, 351–360.^1^ GalNAc-T3 specifically glycosylates the fibroblast growth factor 23 (FGF23), regulating its physiological function. Here we present compelling evidence that FGF23 is a poor substrate of GalNAc-T3, implying that this inefficient glycosylation of FGF23 is the key limiting step in regulating intact biological active FGF23 and phosphate homeostasis.
- González-Ramírez, A. M.; Grosso, A. S.; Yang, Z.; Compañón, I.; Coelho, H.; Narimatsu, Y.; Clausen, H.; Marcelo, F.; Corzana, F.; Hurtado-Guerrero, R. Structural basis for the synthesis of the core 1 structure by C1GalT1. *Nat. Commun.***2022**, *13*, 2398.^2^ The glycosyltransferase C1GalT1 directs a key step in protein O-glycosylation that is important for the expression of the cancer-associated Tn and T antigens. Here, we provide molecular insights into the function of C1GalT1 by solving the crystal structure of the *Drosophila* enzyme–substrate complex.
- Taleb, V.; Liao, Q.; Narimatsu, Y.; García-García, A.; Compañón, I.; Borges, R. J.; González-Ramírez, A. M.; Corzana, F.; Clausen, H.; Rovira, C.; Hurtado-Guerrero, R. Structural and mechanistic insights into the cleavage of clustered *O*-glycan patches-containing glycoproteins by mucinases of the human gut. *Nat. Commun.***2022**, *13*, 4324.^3^ AM0627 is a bis-*O*-glycan mucinase that might work in the last steps of degradation of the mucus, thus providing a source of carbon and nitrogen for *Akkermansia muciniphila*. Here, we provide molecular insights into AM0627 function from X-ray crystallography and computer simulations.
- Bermejo, I. A.; Usabiaga, I.; Compañón, I.; Castro-López, J.; Insausti, A.; Fernández, J. A.; Avenoza, A.; Busto, J. H.; Jiménez-Barbero, J.; Asensio, J. L.; Peregrina, J. M.; Jiménez-Osés, G.; Hurtado-Guerrero, R.; Cocinero, E. J.; Corzana, F. Water Sculpts the Distinctive Shapes and Dynamics of the Tumor-Associated Carbohydrate Tn Antigens: Implications for Their Molecular Recognition. *J. Am. Chem. Soc.***2018**, *140*, 9952–9960.^4^ Here we provide experimental evidence for the conformational differences between the two Tn antigens (GalNAc-α-1-O-Thr/Ser) and confirm the importance of water molecules in the 3D structures explored by each antigen.

## Introduction

**Figure 1:**
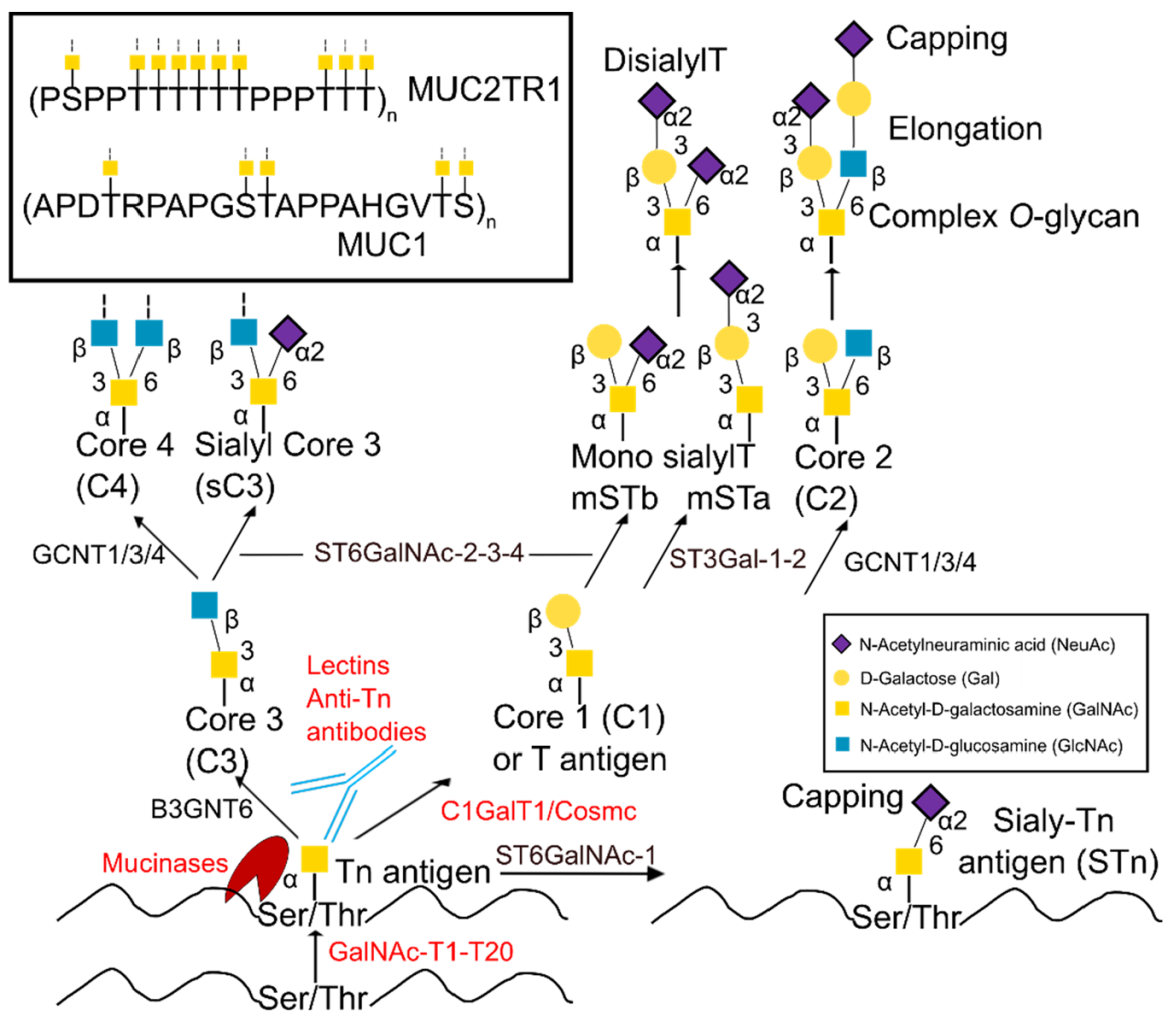
Scheme of the mucin-type O-glycosylation pathway. All of the GTs involved in initiation and core extension are mentioned together with the stereochemistry and glycosidic linkages. The proteins under review in this Account are highlighted in red.

*N*-Acetylgalactosamine (GalNAc) constitutes one of the 10 essential monosaccharides that, when activated as a sugar nucleotide (UDP-GalNAc), is used to build part of the human glycome.^5^ The addition of GalNAc onto Ser/Thr residues (and possibly Tyr) of proteins defines one of the most important, abundant, and complex regulated types of protein O-glycosylation pathways present in eukaryotes, the so-called GalNAc- or mucin-type O-glycosylation.^5−7^ This pathway is chiefly found in the densely clustered, heavily glycosylated domains of mucins and mucin-domain-containing glycoproteins (Figure 1). However, it is also clear that many other proteins contain isolated sites of GalNAc-O-glycosylation, where more than ∼5000 human proteins trafficking the secretory pathway have been identified to date containing one or more mucin-type *O*-glycans.^8^

The O-glycosylation pathway is initiated by a large family of GalNAc-transferases (GalNAc-Ts). These enzymes orchestrate with high fidelity the initial patterns of O-glycosylation on diverse proteins, including the high-density regions in mucins, where 30–50% of the amino acids may be glycosylated.^9^ After the synthesis of the Tn antigen by the GalNAc-Ts, core extension takes place by different glycosyltransferases (GTs). Of particular relevance are C1GalT1, which needs the assistance of the Cosmc chaperone to be functional, and B3GNT6 because they synthesize core 1 and core 3, which act as substrates for core 2 and core 4 production, respectively.^10^ Elongation/branching steps elaborate further the different core structures that are terminated by a capping step (Figure 1).

Mucinases, a type of *O*-glycoprotease that depends on neighboring *O*-glycans for activity, play a fundamental role in degrading mucins as an important step for infectivity^11,12^ or as a nutrient source.^3^ Therefore, mucin-type *O*-glycans can function by acting as a shield and nutrient source for pathogenic microorganisms and commensals/symbionts, respectively,^13^ and have additional roles in protecting proteins from proteolytic cleavage^1,14,15^ and mediating binding of protein receptors toward their protein ligands. Due to these functions, mucin-type *O*-glycans may conceivably interact with almost all physiological processes.^16^ However, the importance of this pathway is not reflected by the single knockouts of *GalNAc-T* genes in mice, which lead to mild or absent phenotypes likely due to the redundant activities between these isoenzymes.^17^ On the contrary, the knockouts of either *C1GalT1* or *Cosmc* are essential at the embryonic level, emphasizing the relevance of this pathway in physiology.^18,19^

At a disease level and particularly in cancer, the controversial relocation of GalNAc-Ts from their usual location in the Golgi apparatus to the endoplasmic reticulum^20,21^ and the well-accepted hypermethylation of the Cosmc promoter leading to its silencing^22^ together with overexpression of ST6GalNAc-I (Figure 1),^23^ have been suggested to be behind of the presence of truncated and aberrant *O*-glycans.^22^ These are tumor-associated carbohydrate antigens (TACAs) found in clinical specimens of different types of cancers, with the most relevant ones being the Tn, T (or core 1, Galβ1-3GalNAc-α-1-*O*-Thr/Ser), and STn antigens. Due to the importance of these TACAs in cancer, numerous antibodies have been developed for cancer treatment and as diagnostic tools.^24^ Lectins such as the human macrophage galactose lectin (MGL) also bind to these TACAs and particularly to Tn antigen, which makes this protein useful for therapeutic applications.^25^

In this Account, we focus on enzymes, antibodies, and lectins (Figure 1) that recognize the GalNAc moiety in glycopeptides. To this end, we will show examples from our laboratories of 3D structures of these proteins in complex with glycopeptides determined by X-ray crystallography or NMR experiments. We will also discuss the commonalities between these different systems in recognition of this important monosaccharide.

### Key Elements in GalNAc-Ts for Substrate Recognition

**Figure 2:**
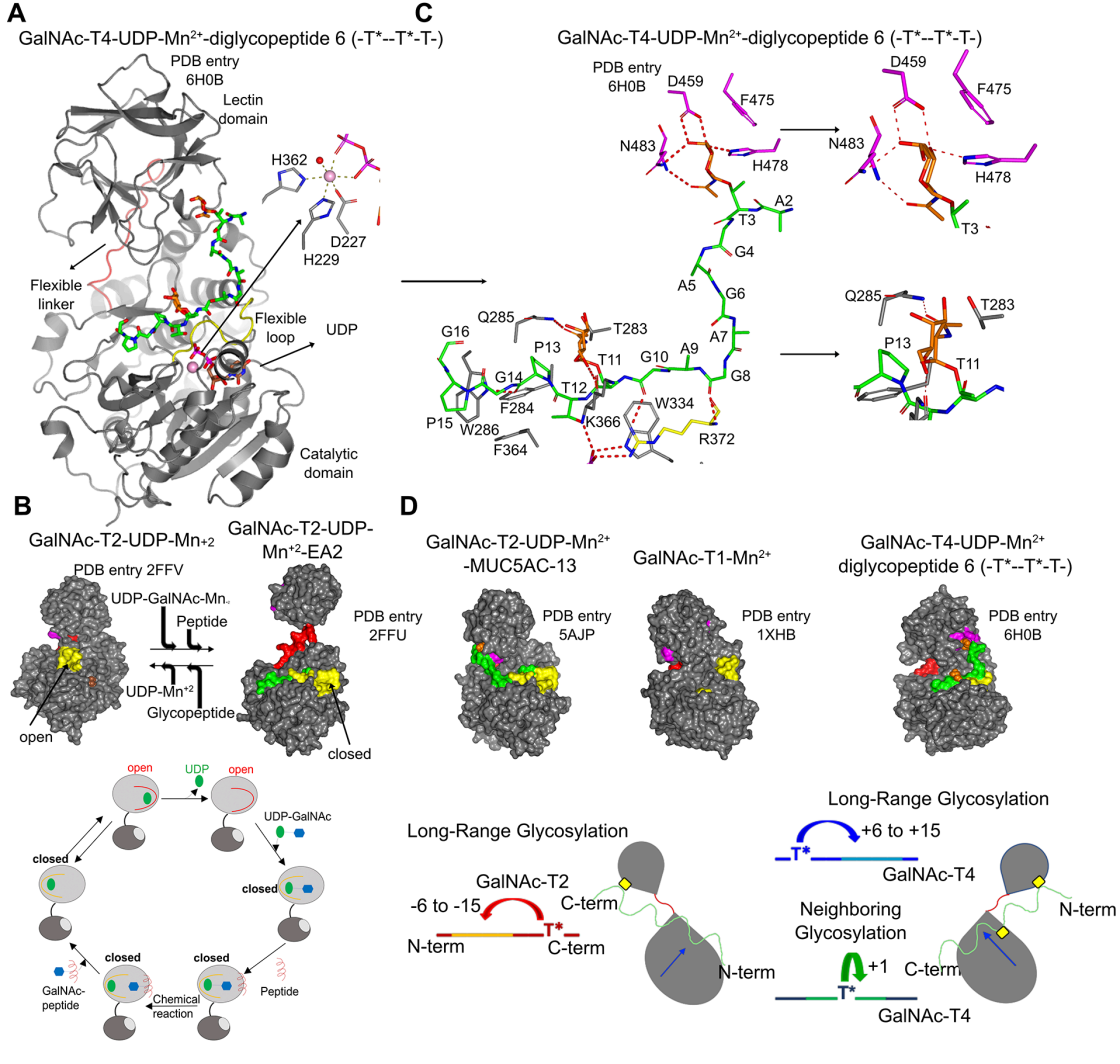
Five structural features define GalNAc-Ts functions. (A) Overall structure of GalNAc-T4. The catalytic and lectin domains are colored in gray, and the interdomain flexible linker is in red. The catalytic domain flexible loop, UDP, the glycopeptides, and the GalNAc moieties are depicted in yellow, brown, green, and orange, respectively. The inset shows the coordination geometry of Mn^2+^. (B) (Upper panel) Surface representation of the inactive form of GalNAc-T2 and the active form of GalNAc-T2. (Lower panel) Catalytic cycle of GalNAc-T2. (C) (Left panel) View of the complete sugar-nucleotide, peptide, and lectin-domain binding sites of GalNAc-T4. (Right panel) Close-up view of the GalNAc-binding sites of the lectin and catalytic domains. The residues of the lectin domain engaged in recognition of the GalNAc moieties are indicated in magenta. (D) (Upper panel) Surface representation of different GalNAc-Ts showing the location of the lectin domain GalNAc-binding sites in the overall structures. Note that GalNAc-T1 has two potential functional GalNAc-binding sites. (Lower panel) Scheme illustrating the different types of O-glycosylation and preferences found for GalNAc-T2 and T4.

GalNAc-T isoenzymes display an N-terminal GT-A fold catalytic domain followed by a C-terminal lectin domain connected by a flexible linker. For optimal binding to UDP-GalNAc, a metal, particularly manganese, is required. This is facilitated by a DxH motif and an additional His residue that coordinates the metal (Figure 2A).^26−28^ Additionally, the catalytic domain contains a flexible loop that adopts two conformations, resulting in either an inactive (open flexible loop) or active form (closed flexible loop) (Figure 2B).^27^ Prior to our work in this field, it was already established how GalNAc-T2 interacts with naked peptides,^27^ but the catalytic mechanism and the molecular basis of how these isoenzymes recognize glycopeptides were unknown.

We managed to trap different structures that enabled us to determine the catalytic cycle of GalNAc-T2.^29^ This, together with our group’s characterization of an inactive GalNAc-T2 (F104S) mutant, which is responsible for low levels of high-density lipoprotein cholesterol in humans,^30^ prompted us to propose a UDP-GalNAc-dependent induced-fit mechanism within an overall BiBi kinetic mechanism (see Figure 2B for details of the mechanism).^31^ Metadynamics calculations of trapped complexes indicated that GalNAc-T2 follows the typical front-face S_N_i-type reaction, where the β-phosphate acts as the catalytic base.^29^ Note that the interactions of GalNAc-T2 with the UDP-GalNAc moiety are discussed in ref (29). Additionally, we hypothesized that the preference of these isoenzymes for glycosylating Thr over Ser residues was due to the Thr methyl group interacting with nearby hydrophobic/aromatic residues.^29^ This was supported by kinetic experiments using different GalNAc-T isoenzymes.^32^

To figure out how these isoenzymes interact with the GalNAc moiety to direct either long-range or neighboring O-glycosylation, we trapped several crystal structures of GalNAc-Ts complexed to different glycopeptides.^1,33−35^ Five features that were indispensable for the recognition of the glycopeptides were revealed. (i) The first feature is the peptide-binding site that is required to recognize the peptide sequences. For example, whereas for the GalNAc-T4-UDP-Mn^2+^-diglycopeptide 6 complex, most of the direct interactions with the peptide were hydrogen bonds (H-bonds) and to a lesser extent hydrophobic interactions (Figure 2C);^33^ for the GalNAc-T2-UDP-Mn^2+^-MUC5AC-13 complex, most of the direct interactions were hydrophobic with few direct H-bond interactions.^34^ Three highly conserved aromatic residues (Phe284, Phe364, and Trp286 in GalNAc-T4) were key residues in the recognition of common peptide motifs such as Pro-X-Pro (X is a small hydrophobic residue) found in acceptor substrates. These examples together with our structures of GalNAc-T3 with glycopeptides illustrated the differences and similarities between these isoenzymes at the peptide-binding site level, which are likely behind the redundancy found for some of the isoenzymes and even the specificity for GalNAc-T3 and GalNAc-T11 toward FGF23^1^ and LA repeats of low-density lipoprotein receptor (LDLR)-related proteins, respectively.^36^ (ii) The flexible loop not only is involved in the catalytic cycle but also contributes to the recognition of the peptide sequences, as seen, for example, for the interactions between Arg372^GalNAc-T4^ with the diglycopeptide 6 (Figure 2C). Of special interest was our study of how the less stable flexible loop closed conformation and the interaction of the acceptor site of a FGF23 glycopeptide with UDP β-phosphate together with the unusual conformation of Trp385 explained why GalNAc-T3 was a poor enzyme on glycosylating FGF23. Note that Trp385, which is conserved between all isoenzymes except in those of the Y family in which the Trp residue is replaced by a Tyr residue,^37^ is highly important in catalysis and also adopts different conformations through the catalytic cycle.^29^ (iii) Isoenzymes such as GalNAc-T4, T7, T10, and T12 were predicted to contain an additional GalNAc-binding site in the catalytic domain to account for their neighboring O-glycosylation capacity.^38^ This was demonstrated by our structure of GalNAc-T4 complexed to the diglycopeptide 6 (Figure 2C). In particular, the GalNAc moiety was mainly recognized by H-bond interactions and to a minor extent by hydrophobic contacts.^33^ We explained why GalNAc-T4 is capable of glycosylating acceptor sites located at +1 with respect to a prior contiguous glycosite (Figure 2D). The molecular basis of the neighboring glycosylation of GalNAc-T12 was further provided, explaining why this isoenzyme prefers to glycosylate acceptor sites located at −1 from a prior glycosite.^39^ For other isoenzymes such as GalNAc-T7 and T10, the molecular basis of how they glycosylate proximal sites from a prior glycosite is not known and merits further investigation. (iv) Our structures also provided the molecular basis of how a distant glycosite from potential acceptor sites can direct long-range O-glycosylation. It is well-accepted that these isoenzymes contain three potential GalNAc-binding sites in the lectin domain. All our structures revealed a single functional GalNAc-binding site, located on opposing sides of the lectin domain when the overall structures were superimposed (Figure 2C and D). The latter clearly explained why isoenzymes such as GalNAc-T2 had different long-range glycosylation preferences to those of GalNAc-T3 or T4 (see Figure 2D). Although it was demonstrated that the peptide sequences around the O-glycosite influenced O-glycosylation in a lectin-dependent manner,^40^ in all of our structures the GalNAc moiety was mainly the only feature recognized by hydrogen bonds and CH-π interactions by mostly conserved residues of the lectin domain (Figure 2C). (v) Finally, to solve why the lectin GalNAc-binding site was located in different sides of the lectin domain depending on the isoenzyme, we conducted molecular dynamics (MD) simulations of GalNAc-T2 chimeras and kinetics experiments that revealed that the flexible linker was responsible for the location of the GalNAc-binding site in the lectin domain. This exemplified for the first time that the flexible linker was responsible for the location of the GalNAc-binding site in the lectin domain, which clearly is behind the different long-range glycosylation preferences.^35^ A previous study from our group further demonstrated that the flexible linker is also responsible for the dynamics of these isoenzymes, explaining why they can adopt compact and extended forms in solution.^34^

Nevertheless, more experiments are needed to determine, for example, how GalNAc-T11 glycosylates LA repeats or GalNAc-T7/T10 to achieve multiple O-glycosylations mainly in mucins. Other functions such as the hierarchical functions of these isoenzymes on complex substrates such as MUC1 has been revealed recently by using a combination of NMR and computational experiments.^41^

### Molecular Recognition of GalNAc *O*-Glycans by C1GalT1

**Figure 3:**
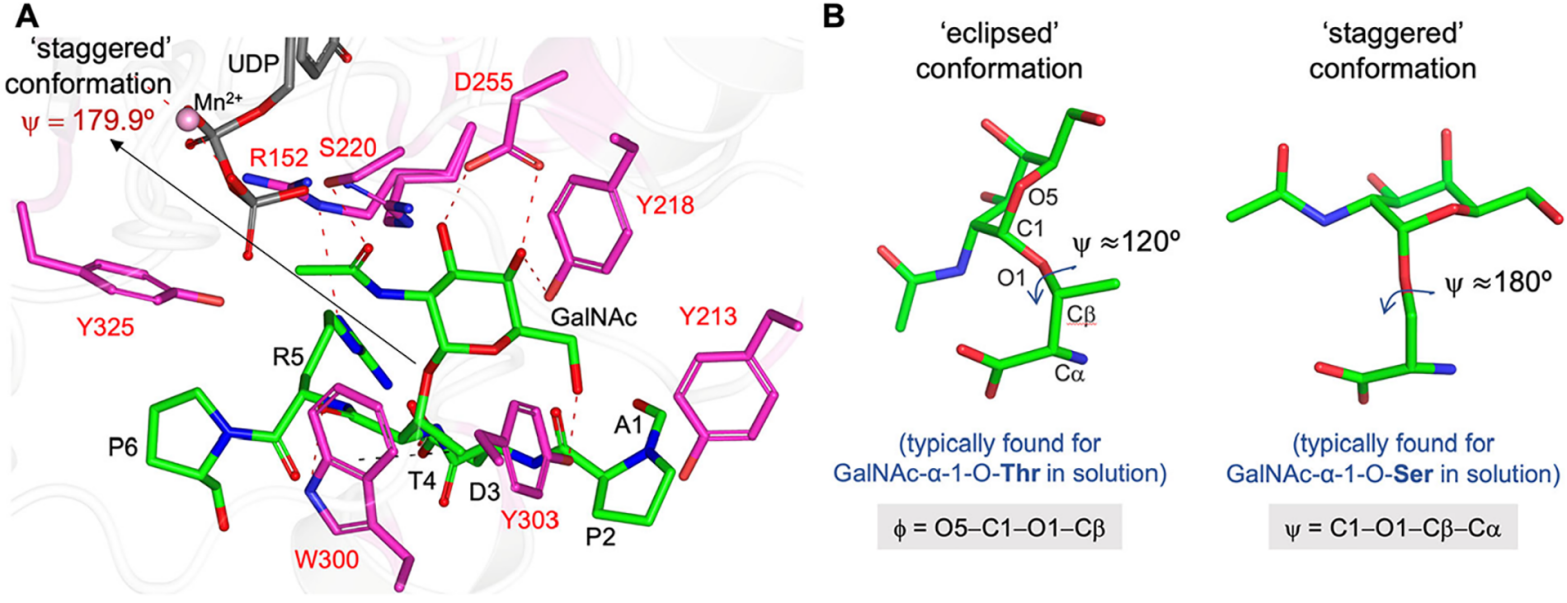
Molecular recognition of glycopeptides by C1GalT1. (A) Close-up view of the active site for the *Dm*C1GalT1-UDP-APDT*RP complex (PDB entry 7Q4I). The carbon atoms of the residues interacting with the glycopeptide are shown in magenta. The UDP carbon atoms are shown in gray. (B) Main conformation found in solution for the Tn antigen with Thr GalNAc-α-1-*O*-Thr (left panel) and for the Tn antigen with Ser GalNAc-α-1-*O*-Thr (right panel). The CH-π interaction between the methyl group of Thr4 and Trp300 is shown by the black dashed line.

We have recently reported the X-ray structure of *Drosophila melanogaster* C1GalT1 (*Dm*C1GalT1), which does not appear to require a chaperone for expression, in complex with the MUC1-related glycopeptide APDT*RP (* denotes GalNAc).^2^ This enzyme is responsible for adding a galactose at carbon 3 of GalNAc through a β-O-glycosidic linkage to form core 1. The GalNAc moiety of the glycopeptide forms several H-bonds with the enzyme, while the peptide sequence is recognized by hydrogen bonds and CH-π interactions (Figure 3A). Thus, in contrast to the lectin domain of GalNAc-Ts described earlier, in this enzyme the sugar does not establish CH-π interactions with the aromatic residues.

The crystal structure shows that the glycosidic bond of α-GalNAc-Thr has a staggered conformation (torsion angle ψ ≈ 180°, Figure 3B) that does not occur in aqueous solution for the Tn-Thr-containing glycopeptides but is exhibited by the Ser variant.^4,42^ MD simulations performed for *Dm*C1GalT1 in complex with UDP and APDT*RP glycopeptide also predicted the staggered conformation for the GalNAc-α-1-*O*-Ser moiety and demonstrated that the eclipsed conformation fixed in α-GalNAc-Thr displayed a loss of interactions between the peptide and the enzyme compared to those found in the X-ray structure. On this basis, we hypothesize that C1GalT1 recognizes a high-energy conformation of the GalNAc-α-1-*O*-Thr linkage that somehow compensated for the additional CH-π interaction observed for the methyl group of Tn-Thr (see black dashed line in Figure 3A between this group and Trp300). As a result, the enzyme exhibits a comparable affinity for both Tn-Ser and Tn-Thr.

### Molecular Recognition of GalNAc *O*-Glycans by Mucinases

The enzymatic action of mucinases depends on prior recognition of an *O*-glycopeptide by the enzyme, and GalNAc is a crucial moiety for this purpose. Only five mucinases (AM0627, BT4244, IMPA, ZmpB, and ZmpC) have been fully characterized to date. The different structural characteristics and recognition preferences reveal a notable capacity to adapt to the diverse patterns of the glycopeptides, suggesting an appreciable tolerance for the structure of the *O*-glycopeptide. On the other hand, mucinases share several similarities with conservation of various residues in the active site, but they vary for others that are responsible for the recognition preferences and specificity of the enzyme.

**Figure 4:**
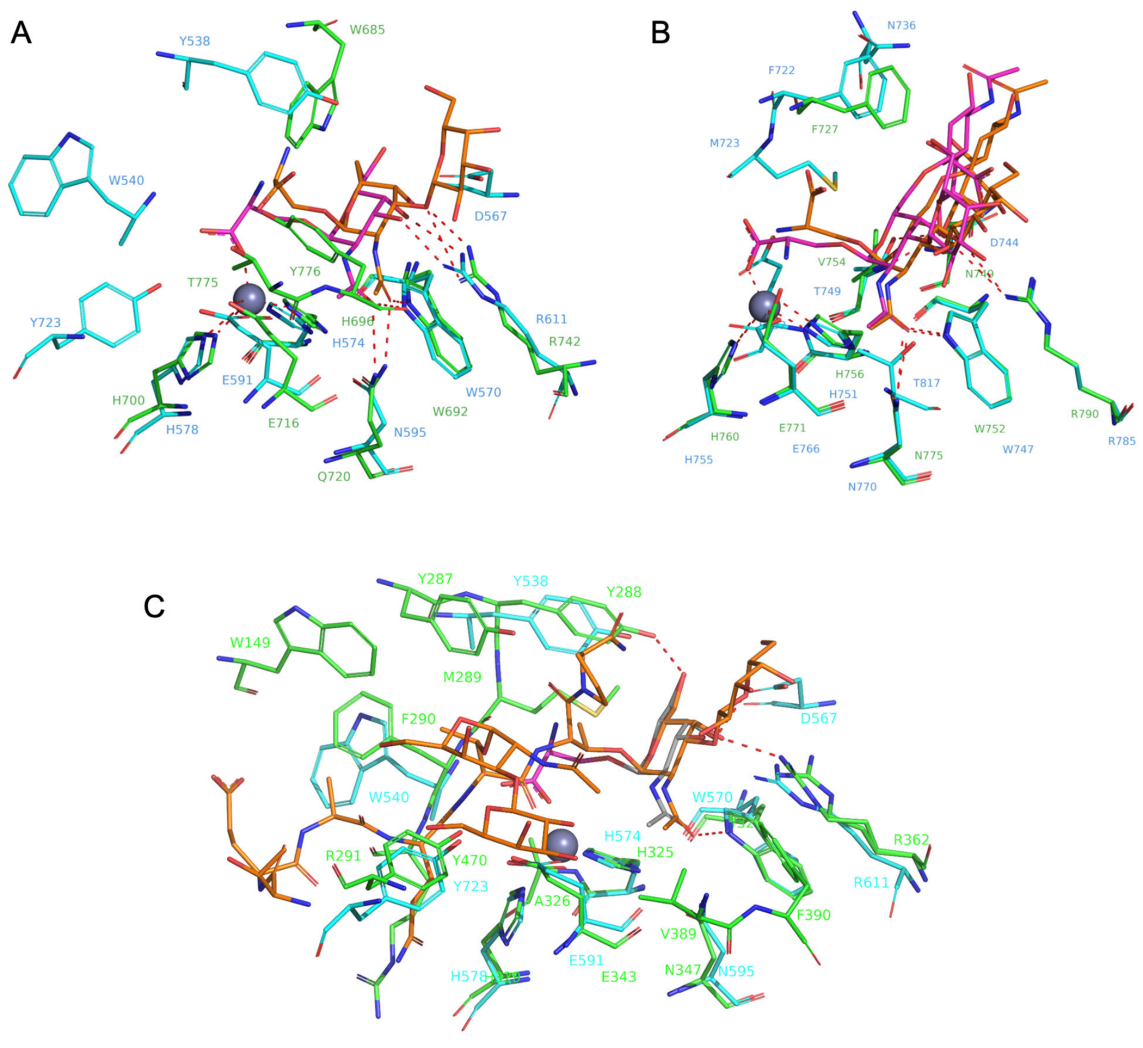
Comparison between the different active sites of mucinases. (A) Superimposition of the active sites of IMPa (green) and BT4244 (cyan) showing main interactions with the sugar units of the *O*-glycopeptides (orange for IMPa and magenta for BT4244). (B) Superimposition of the active sites of ZmpB (green) and ZmpC (cyan) showing main interactions with the corresponding *O*-glycopeptides (orange for ZmpB and magenta for ZmpC). (C) Superimposition of the active sites of complexes formed between BT4244 (cyan) and AM0627 (green) with a Tn *O*-glycan (magenta) and bis-T-glycopeptide (orange), respectively. PDB entries for the structures of AM0627, IMPa, BT4244, ZmpB, and ZmpC are 7SCI, 5KDX, 5KD8, 5KDU, and 6XT1, respectively.

Common features to all mucinases regarding recognition of GalNAc comprise H-bonds of the *N*-acetyl group with a Trp and with the backbone of an Asn (or Gln for IMPa) as well as one or two H-bonds between the hydroxyl groups and an Arg. All mucinases have an additional aromatic residue (Phe, Trp, or Tyr) presenting different interactions with GalNAc that could be responsible, in part, for the selectivity to different *O*-glycopeptides (Figure 4). A residue of Tyr leads to a H-bond with a hydroxyl group of GalNAc in AM0627 and BT4244,^13,43^ while in ZmpB and ZmpC a residue of phenylalanine forms a CH-π interaction with the sialic acid. These differences represent the ability of mucinases to accept different *O*-glycopeptides as mentioned earlier, but with restricted substrate specificities when necessary.^44^

Whereas ZmpB, ZmpC, and IMPa only recognize monoglycosylated peptides, mucinases like AM0627 and BT4244 are capable of recognizing bis-*O*-glycans through Tyr470 (AM0627) and likely Tyr723 (BT4244) by forming H-bond interactions with O6 of GalNAc and CH-π interactions with GalNAc. The importance of this interaction has been experimentally confirmed to be essential for their activity.^3^

### Molecular Recognition of GalNAc by Antibodies

Anti-MUC1 and anti-Tn antibodies have long been used clinically in cancer diagnosis and therapy.^24,45^ Initial efforts were focused on IgG1 antibodies that block MUC1 on the surface of cancer cells. However, anti-MUC1 antibodies have also been conjugated to radioisotopes to use in both imaging and cancer therapy, as well as to small-molecule toxins, resulting in antibody–drug conjugates (ADCs) approved for the treatment of various cancers, including trastuzumab emtansine, brentuximab vedotin, and gemtuzumab ozogamicin. The hypothesis that cancer-specific antibodies can be fused with anti-CD3 antibodies to reverse T-cell-mediated killing in cancer patients has also led to the approval of catumaxomab and blinatumomab. On the other hand, several anti-MUC1 antibodies have been tested for CAR-T cells, such as HFMG-2, SM3, and more recently 5E5.^46^ These anti-MUC1 antibodies mainly recognize the peptide fragment, but glycosylation of the peptide epitope generally enhances binding (see below). In some cases, the carbohydrate either promotes proper presentation of the peptide or makes additional contact with the protein. In other cases, the antibody may accurately recognize the GalNAc (or sugar) component, representing the primary epitope binding.^47^

**Figure 5:**
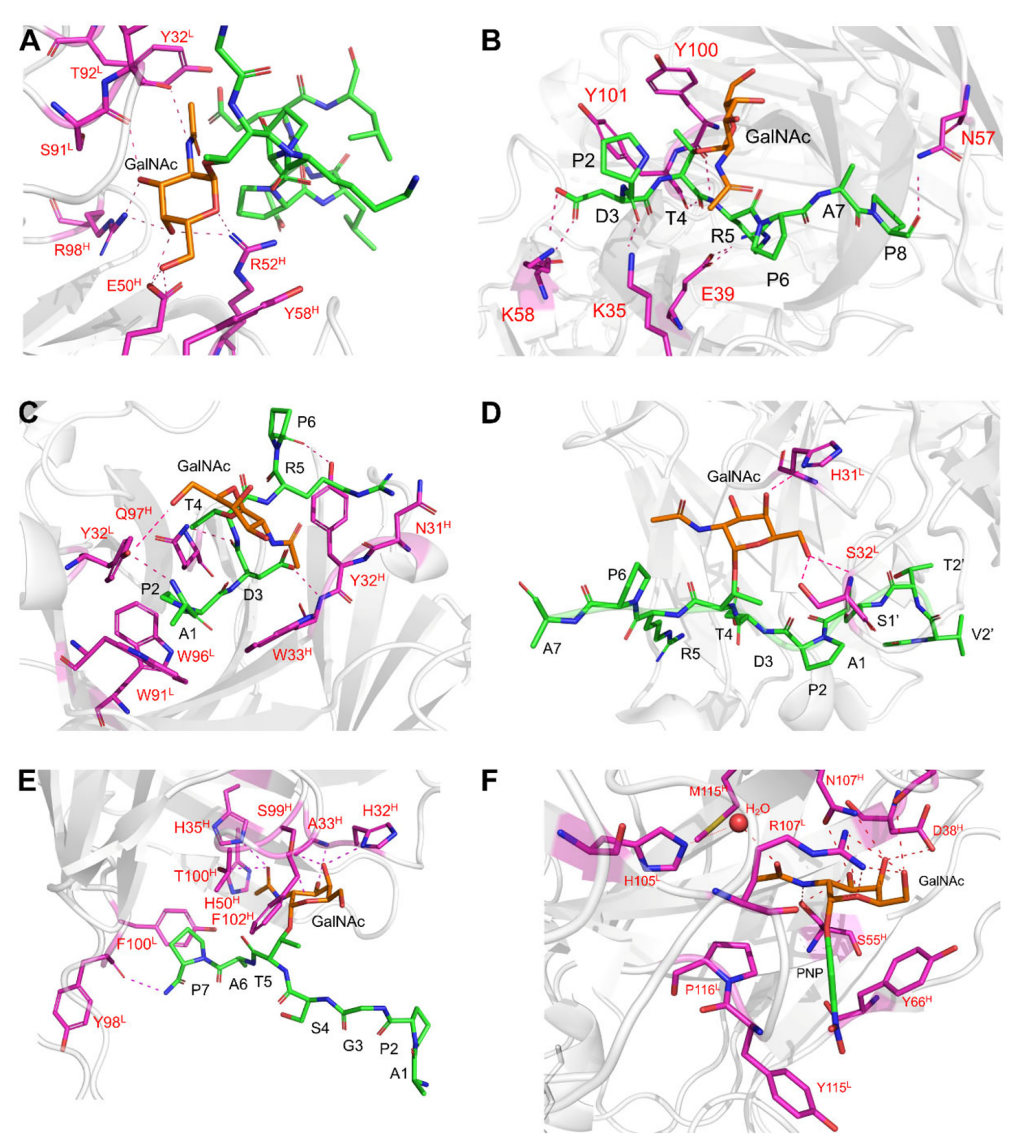
Molecular recognition of Tn-glycopeptides by antibodies. (A) Close-up view of the binding site of the 273 mAb bound to the ERGT*KPPLEEL glycopeptide, showing the interactions of the protein with the GalNAc unit (PDB entry 3IET). (B) Close-up view of the binding site of the AR20.5 antibody and the glycopeptide APDT*RP (PDB entry 5T78). (C) Close-up view of the binding site of the SM3 antibody and glycopeptide APDT*RP (PDB entry 5A2K). (D) Close-up view of the binding site of the SN-101 antibody and a MUC1-like glycopeptide (PDB entry 6KX1). Only the interactions between the protein and the GalNAc unit are shown. (E) Close-up view of the binding site of the antibody 5E5 and glycopeptide APGST*AP (PDB entry 6TNP). (F) Close-up view of the binding site of the antibody G10C and αGalNAc-PNP (PDB entry 7UT3). Note that in all panels the residues of the antibody engaged in recognition of the glycopeptides and/or the GalNAc moieties are indicated in magenta.

The first high-resolution X-ray structure that shed some light on the role of the GalNAc residue in recognition processes between antibodies and a Tn-glycopeptide was reported for a complex with the 273 antibody and shows that each hydroxyl group of GalNAc forms at least one hydrogen bond with the antibody (Figure 5A).^48^ The NH and methyl groups of the acetamide interact via a hydrogen bond and a CH-π interaction with the antibody. The peptide fragment is involved in several hydrogen bonds, and the two Pro residues of the sequence are engaged in CH-π contacts. Of note, the glycosidic linkage of the GalNAc-α-1-*O*-Thr residue displays the eclipsed conformation found for the Tn-Thr antigen in solution.^4^

The X-ray structure of a MUC1-like peptide and the corresponding Tn-glycopeptide bound to the AR20.5 antibody (Figure 5B) underwent a successful phase I clinical trial, showing that the GalNAc moiety has no specific contacts with the antibody.^49^ The higher affinity for the glycopeptide compared to the parent peptide indicates that the GalNAc moiety improves the shape compatibility of the peptide with the antibody. Again, the conformation of the glycosidic linkage between GalNAc and Thr recognized by the antibody adopts the eclipsed conformation. Our group provided the first crystal structure of the Tn-glycosylated MUC1 epitope (APDT*RP) with antibody SM3 (Figure 5C).^50^ The crystallographic analysis of the structure with the Tn-Thr glycopeptide showed that the GalNAc motif does not significantly affect the conformation of the peptide backbone in the SM3-bound state. The glycosidic linkage of the glycopeptide adopts the expected exoanomeric/syn conformation with an eclipsed arrangement.^4^ This geometry allows the formation of an intermolecular hydrogen bond between the hydroxymethyl group of GalNAc and the antibody, and the *N*-acetyl group of this sugar stacks with the aromatic ring of Trp33^H^, providing the impetus for the observed selectivity of SM3 for GalNAc-containing antigens. In sharp contrast, the GalNAc moiety in the bound state of the Tn-Ser and Tn-Cys glycopeptides with the SM3 antibody^50^ does not establish stabilizing contacts with the protein, and the glycosidic linkage displays a high-energy conformation that is not populated in solution. These results may explain the higher affinity of the SM3 antibody for the glycopeptide with Tn-Thr.^50^

The epitope mapping of a MUC1 library containing peptides and Tn-glycopeptides with several glycosylation sites and two families of cancer-related antibodies has been recently characterized.^51^ The first family includes two anti-MUC1 antibodies, VU-3C6 and VU-11E2, that recognize tumor-associated MUC1 in breast cancer. The second group comprises two anti-Tn antibodies, 8D4 and 14D6. In all cases, the H2 proton and the *N*-acetyl moiety of GalNAc are in contact with the antibody. Interestingly, NMR data suggest that 14D6 and 8D4 antibodies prefer binding to the Tn-Ser over the Tn-Thr. The extra flexibility of the GalNAc-Ser pair may allow the glycopeptide to display the required conformation in the bound state without a significant entropy penalty.

The crystallographic analysis of the antibody SN-101 bound to a Tn-glycopeptide related to MUC1 (Figure 5D)^52^ shows that the antibody interacts with the sugar through only two H-bonds. The Tn-Thr antigen displays the glycosidic linkage’s characteristic eclipsed conformation. Related to this, our group has described the X-ray structure of antibody 5E5 with a Tn-glycopeptide derived from APGSTAP, which recognizes the entire GalNAc unit as a primary epitope (Figure 5E).^53^ The X-ray structure reveals that all hydroxyl groups of GalNAc, except OH6, are engaged in hydrogen bonds with the antibody. The carbonyl group of the sugar is involved in two hydrogen bonds, and the methyl group is engaged in a CH-π interaction with His50^H^. The lack of interactions with the hydroxymethyl group explains why this antibody can recognize STn-containing glycopeptides.^54^ In addition, the serine residue is exposed to the solvent, which allows the antibody to interact with the diglycosylated peptide. The glycosidic linkage of the glycopeptide in complex with the antibody adopts the typical eclipsed conformation.^4,55^ The peptide moiety forms only one hydrogen bond with the protein in the C-terminal region, allowing some degree of promiscuity. Indeed, our studies and others indicate that the antibody might interact with glycopeptides that comprise the −T*–X–P– motif, which is present in the MUC1 tandem-repeat sequence but also in many other proteins.

Antibody G10C, which recognizes GalNAc bound to the side chain of Tyr in natural peptides and proteins with high affinity and selectivity, has been recently developed (Figure 5F).^46^ The X-ray structure of G10C-Fab in complex with a GalNAc-Tyr mimic shows that hydroxyl groups and the endocyclic oxygen form hydrogen bonds with the antibody. The hydrogen bonds involving the *N*-acetyl group explain the selectivity toward this sugar.

### Molecular Recognition of GalNAc by Lectins

**Figure 6:**
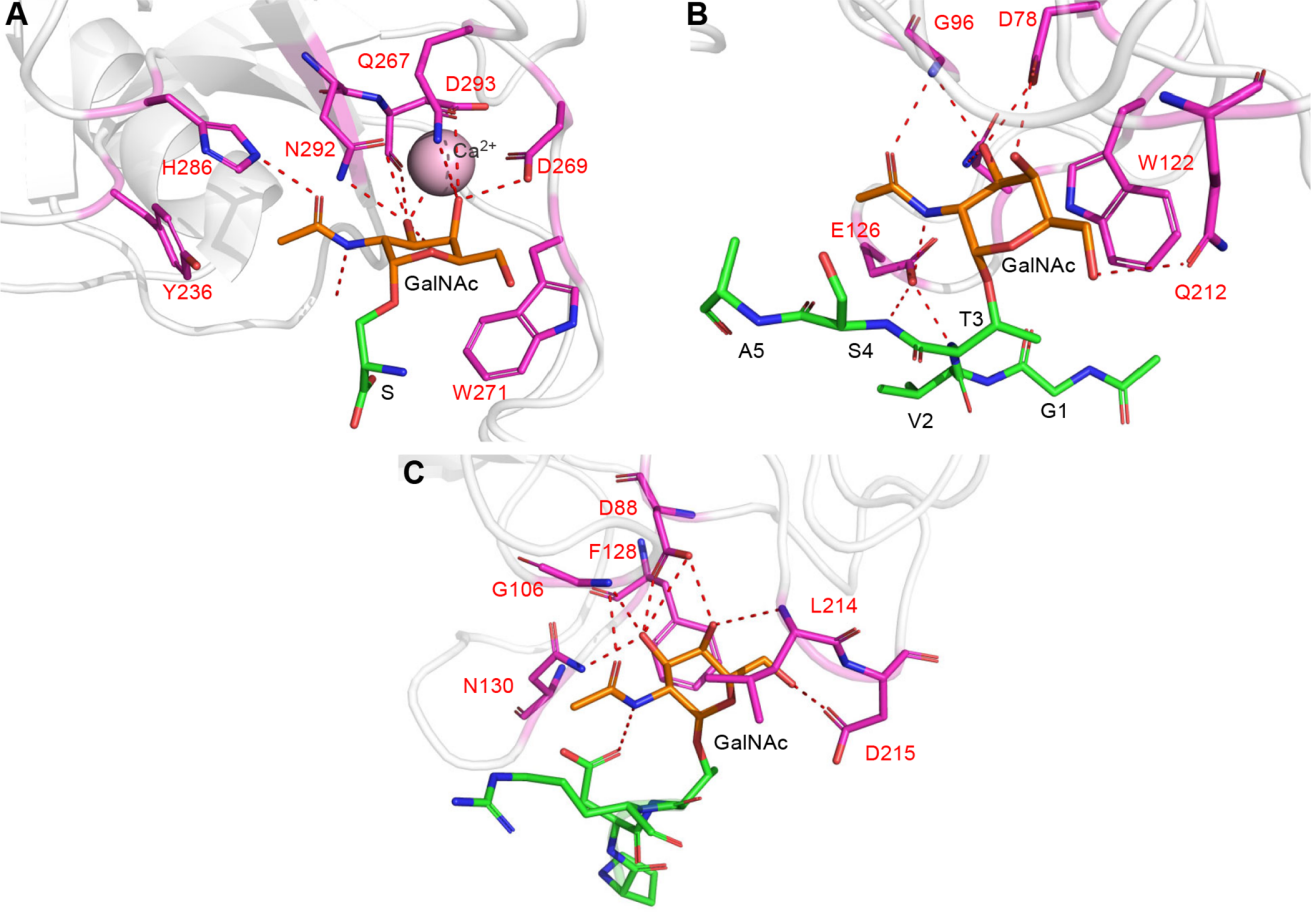
Molecular recognition of Tn-glycopeptides by lectins. (A) Close-up view of the binding site of the lectin hMGL bound to Tn-Ser (PDB entry 6W12). (B) Close-up view of the binding site of the *Bauhinia forficata* lectin bound to a Tn-containing glycopeptide derived from MUC1 (PDB entry 5T5O). (C) Close-up view of the binding site of SBA lectin bound to PDT*R glycopeptide (PDB entry 4D69). Note that in all panels the residues of lectin engaged in the recognition of the glycopeptides and/or the GalNAc moieties are indicated in magenta.

In the context of cancer, various lectins that bind to Tn-glycopeptides have been successfully employed as biomarkers^56^ and cancer progression and anticancer agents.^57^ The binding mode in the human macrophage galactose lectin (hMGL) bound to a GalNAc residue, and the Tn-Ser antigen (Figure 6A)^58^ is nearly identical for both substrates, with the vicinal groups OH3 and OH4 coordinated with the protein and a structural calcium ion, confirming that this is a Ca^2+^-dependent (C-type) lectin. The carbonyl group of the acetamide is hydrogen-bonded with the side chain of His286, and its methyl group and the hydrophobic face of the sugar are involved in CH-π interactions. Regarding lectins as biomarkers, there are some other interesting X-ray structures. For example, complexes with the Tn-Ser antigen have been described for *Helix pomatia agglutinin* (HPA),^59^ SNA-II,^60^ winged bean lectin (*Psophocarpus tetragonolobus*),^61^ and *Vatairea macrocarpa* (VML).^62^ In these cases, all hydroxyl groups as well as the oxygen of the *N*-acetyl of the sugar are involved in hydrogen bonds with the residues of the lectin. Except for HPA, CH-π interactions occur with aromatic residues (Phe or Trp) on the α-face of the sugar. Remarkably, the underlying Ser does not interact with the lectin, except at SNA-II. In all of these complexes, the Tn-Ser antigen exhibits a staggered conformation. Recently, the complex between *Bauhinia forficata* lectin and Tn-containing glycopeptides was reported.^63^ The binding of the GalNAc residue and the lectin is shown in Figure 6B. It is noteworthy that the carboxylate group of Glu126 forms hydrogen bonds with both the *N*-acetyl of GalNAc and the NH of glycosylated threonine, promoting recognition of this MUC1-derived fragment.

We have reported that the peptide sequence flanking the Tn antigen can modulate Tn recognition by two plant lectins (soybean agglutinin (SBA) and Vicia villosa B-4 agglutinin (VVA)) that show higher affinity for the Tn antigen when incorporated into the most immunogenic peptide region of mucin MUC1 (sequence PDTR).^64^ The charged residues Arg and Asp in this sequence provide relevant interactions with the lectin surface likely favoring the presentation of the sugar moiety to the lectin. We could determine by X-ray of SBA lectin bound to the glycopeptide PDT*R (Figure 6C).^64^ Interestingly, while two different binding modes were observed for the peptide, the GalNAc unit exhibits the same interaction pattern in both modes. GalNAc-lectins can also discriminate between glycopeptides containing the Tn-Thr or the Tn-Ser, which can be explained by the different conformational behavior and dynamics of these two Tn units.^65^

hMGL and several plant lectins bind with high affinity to GalNAc-Tyr.^66^ Saturation transfer difference (STD) NMR experiments between this lectin and a small glycopeptide containing this moiety show that this lectin is in close contact with H1, H4, and the acetamide group of the GalNAc residue, with weaker STD signals observed for the remaining protons. These results indicate close interactions between hMGL and the entire GalNAc residue of the glycopeptide.

## Differences and Commonalities between Proteins in Recognizing *O*-GalNAc Glycans

Molecular recognition of GalNAc-containing glycopeptides strongly depends on the recognition of the sugar moiety, with the exception of some anti-MUC1 antibodies. However, focusing exclusively on the GalNAc unit, a certain level of degeneracy is observed within the same families of the proteins. The flexibility of the substrate might facilitate the variability in the interactions, allowing changes in crucial residues. An example of this situation is observed in ZmpB,^67,68^ which has a pocket presenting a considerable basic surface complementary with the acidic carboxylate of a sialic acid unit present in the glycopeptide. The presence of two additional subsites for the sialic acid stabilizes the primary interactions with GalNAc at the active site. A similar situation takes place with mucinase IMPa, which has an open region that is susceptible to forming π-interactions with a Trp, where AM0627 and BT4244 have a Tyr and an Asp residue that interact with 6-OH of GalNAc, blocking recognition of substrates with substituents at that position.

In GalNAc-Ts, the lectin domain presents equivalent residues, leading to similar interactions.^6,34^ While most H-bonds and CH-π interactions are conserved for several GalNAc-Ts (see Figure 2C), some nonconserved interactions were observed, such as those found in GalNAc-T3.^1^

Some antibodies, such as SM3, present hydrogen bonds between a residue of Tyr and the hydroxymethyl group of GalNAc, and its methyl group interacts weakly through a CH-π contact with a Tyr residue (see, e.g., PDB entries 5A2K,^48^5OWP,^69^ and 6FRJ(71)). For antibody 237-mAB, a stacking interaction between the methyl group of the *N*-acetyl moiety and a Trp residue was also found.^50^

**Figure 7:**
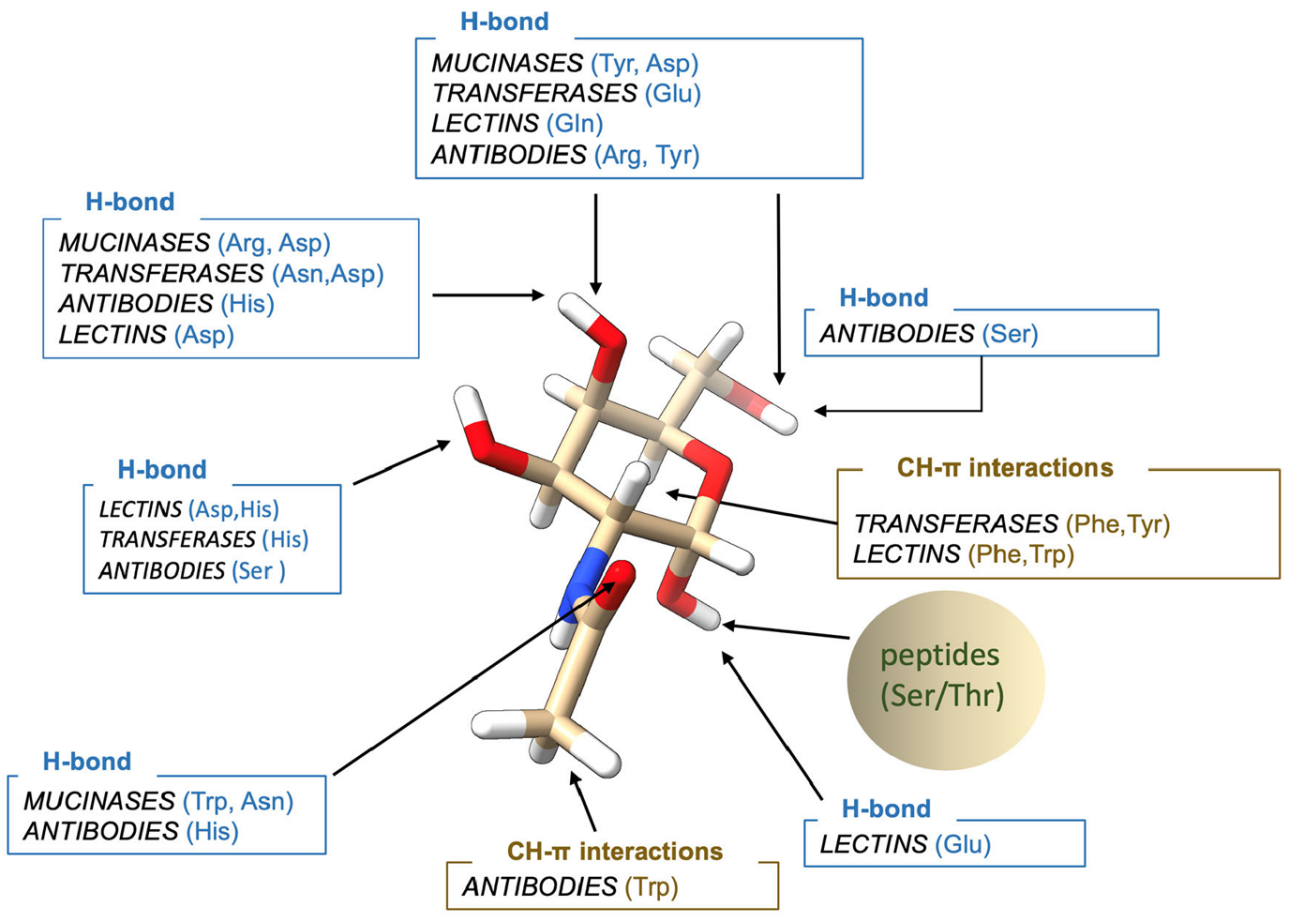
Main common recognition modes for the different families of proteins.

When all of these proteins are considered together, despite the commonalities that are found inside each family of proteins (Figure 7), there is not an observable pattern between the different families, other than some common residues like Tyr, His, or Asp, which are responsible for hydrogen-bond interactions. This is not surprising because of the different functions exerted by mucinases, glycosyltransferases, antibodies, and lectins. In antibodies the role of GalNAc is not so crucial as in other cases, and recognition is limited to a minimum, although there are some exceptions, such as those found for the 5E5 antibody. In contrast, the affinity of lectins by their ligands is determined by their recognition to the GalNAc moiety by a series of hydrogen bonds, as well as CH-π interactions.

## Conclusions and Future Perspectives

This Account demonstrates the important role that the GalNAc moiety plays in the molecular recognition of relevant glycopeptides, such as those that are hallmarks of cancer, by their corresponding biological targets, such as lectins and antibodies. Moreover, GalNAc is an active bystander involved in the synthesis and degradation of *O*-glycans by GTs and mucinases, respectively, by assisting these enzymes in localizing the glycopeptide in the correct position. At the interaction level, GalNAc is recognized by several hydrogen bonds involving its hydroxyl groups, especially OH3 and OH4, located in the same plane, as well as CH-π interactions, engaging the hydrophobic alpha side of the sugar or the −CH_2_– of the hydroxymethyl group. In general, selectivity toward this carbohydrate is achieved through hydrogen bonds with the *N*-acetyl group of GalNAc, which in some cases is complemented by CH-π interactions via the methyl group. The structural information on GalNAc recognition described in this work may be useful in developing drug-design strategies for GTs or mucinases to treat diseases in which they are involved. In addition, the information obtained with antibody and lectin complexes may be a promising tool for developing more effective cancer vaccines as well as tools for cancer diagnosis.
